# A comparison of routine and case-managed pathways for recovery from musculoskeletal disorders in people in employment

**DOI:** 10.1080/09638288.2021.1912837

**Published:** 2021-04-25

**Authors:** Beverly P. Bergman, Evangelia Demou, James Lewsey, Ewan Macdonald

**Affiliations:** a Institute of Health and Wellbeing, University of Glasgow, Glasgow, UK; b MRC/CSO Social and Public Health Sciences Unit, University of Glasgow, Glasgow, UK

**Keywords:** Musculoskeletal disorder, case management, intervention, sickness absence, rehabilitation, health inequalities

## Abstract

**Purpose:**

To compare outcomes in employed people from an enhanced routine management pathway for musculoskeletal disorders within National Health Service Scotland with an existing active case-management system, Working Health Services Scotland.

**Materials and methods:**

The study comprised a service evaluation using anonymised routinely collected data from all currently employed callers presenting with musculoskeletal disorder to the two services. Baseline demographic and clinical data were collected. EuroQol EQ-5D™ scores at the start and end of treatment were compared for both groups, overall and by age, sex, socio-economic status, and anatomical site, and the impact of mental health status at baseline was evaluated.

**Results:**

Active case-management resulted in greater improvement than enhanced routine care. Case-managed service users entered the programme earlier in the recovery pathway; there was evidence of spontaneous improvement during the longer waiting time of routine service clients but only if they had good baseline mental health. Those most disadvantaged through mental health co-morbidity showed the greatest benefit.

**Conclusions:**

People with musculoskeletal disorders who have poor baseline mental health status derive greatest benefit from active case-management. Case-management therefore contributes to reducing health inequalities and can help to minimise long-term sickness absence. Shorter waiting times contributed to better outcomes in the case-managed service.

## Introduction

It has been estimated that 14% of all GP consultations are for musculoskeletal disorders [1], resulting in the loss of 10 million working days annually across the UK [2]. In 2008, the overall cost of being out of the workforce due to ill-health was greater than the annual budget for the National Health Service (NHS) [3]. Mental health conditions, especially depressive disorders, commonly co-exist with musculoskeletal disorders [4–6] and are increasingly impacting on ability to work [7]. Biopsychosocial factors, especially the psychosocial component, are important in determining outcomes, from the acute phase onwards [8], and persistent depressive symptoms are associated with poor return to work outcomes [9]. Long-term sickness absence has a disproportionately high impact, accounting for over one-third of all days lost and 75% of the costs of sickness absence [10]. Preventing or minimising long-term sickness absence due to musculoskeletal disorders is therefore an important component of strategies to reduce working days lost. In 2006, the UK Health & Safety Executive published a research report which concluded that there was good evidence to support active case management of employees with musculoskeletal disorders, using a biopsychosocial model, as a cost-effective approach to recovery and return to work [11]. Nonetheless, few people with musculoskeletal symptoms are able to benefit from such support, which is not routinely available through the NHS. Whilst the NHS aspires to provide high-quality clinical care to all, which is free at the point of delivery, it is generally only the larger employers who are able to provide a comprehensive occupational health service which includes case management; employees of small to medium enterprises (SMEs) and the self-employed are unlikely to have access to such services.

The traditional model of management of musculoskeletal conditions in the UK follows a pathway of initial presentation to a general practitioner or accident and emergency department, with onward referral to a physiotherapist or appropriate specialist as necessary. For non-urgent conditions, waiting list delays can be substantial, potentially leading to prolonged absence from work and perhaps worsening of the condition. In 2011, as part of the redesign of Scotland’s Allied Health Professional (AHP) Musculoskeletal Services (MSK), NHS Scotland introduced a pilot MSK National Advice and Triage Service (MATS) aimed at improving the management of non-emergency musculoskeletal disorders [12]. The service operated at three levels; self-management, accelerated access to AHP care via a central “MSK hub”, and referral to an existing case-managed service, Working Health Services Scotland (WHSS) aimed at self-employed people or those working within enterprises employing less than 250 people [13,14]. Case management involved referral of the patient to a trained case manager, who acts as a coordinator for the care pathway, facilitating access to services and monitoring progress. The case management approach of WHSS followed the holistic biopsychosocial model [11,15] as distinct from the more traditional medical triage model of MATS. WHSS aimed to assess and offer treatment within 5−10 days of referral, as opposed to the routine NHS waiting times of the MSK service.

Patients were entered into the MATS pilot if they lived within the catchment area of NHS Lanarkshire and telephoned NHS 24, the Scottish NHS 24-hour helpline, with a muscle or joint problem not warranting urgent A&E referral. Following telephone triage, callers were either:

- referred for medical assessment;
- directed to self-management resources;
- directed to self-management resources, and their local MSK hub, or;
- directed to self-management resources, their local MSK hub, and WHSS.

Direct entry to WHSS was also available to those in eligible employment categories via general practitioners, NHS physiotherapists or other health professionals, or by self-referral, in all Health Board areas [16].

The aim of this paper is to compare outcomes in employed people directed to the MSK hub alone with those undergoing rapid-access active case management for musculoskeletal disorders by WHSS, using change in EQ-5D score as the measure of outcome, and to assess the impact of mental health status at baseline.

## Methods

### Participants and sample size

Participants were all users of the NHS MSK service and WHSS who met the eligibility criteria of employment status and diagnostic classification described below. The sample size was constrained by the client base for each service; 100% of contacts were recorded. Baseline data were routinely collected for each caller to the services, including date of birth (WHSS) or age at first contact (MSK), gender, employment status, nature and duration of problem, dates of referral and first assessment, and referral details. For WHSS, age at first contact (in years) was calculated from date of birth and date of enrolment. Time to commencement of treatment was calculated as time in days from enrolment to first assessment. This assessment was made at first contact with a health professional and treatment was deemed to commence at this point. Data were collected between March 2010 and March 2014 for WHSS, and between May 2012 and March 2014 for MSK.

### Socio-economic status

Socio-economic status (SES) was derived from the postcode of residence, using the Scottish Index of Multiple Deprivation (SIMD). SIMD is calculated on a regional basis, in datazones having a mean population of 800, based on income, employment, health, education (including skills and training), housing, crime, and access to services [17]. SIMD has been used to derive quintiles of SES for the Scottish population, ranging from 1 (most deprived) to 5 (least deprived). We used postcode of residence to categorise the study participants according to the general population quintiles.

### Diagnostic classification

“Musculoskeletal disorder” was recorded at the discretion of the call handler or case manager in consultation with the service user, and was not defined by ICD code. As the WHSS dataset encompassed contacts for a wide range of conditions and the aim of the study was to compare WHSS with the routine NHS MSK service, for the purposes of this study the data were filtered to include only musculoskeletal disorders. Only people currently in employment (whether or not on sick leave) were included in the study.

### Outcome measures and statistical analysis

The EuroQol Questionnaire (EQ-5D™) is a standardised multidimensional assessment tool for measuring generic health status. The five dimensions measured are mobility, self-care, usual activities, pain and discomfort, and anxiety and depression. The questionnaire is augmented with a visual analogue scale (EQ-VAS). The five dimensions are used to generate a single weighted index score [18]. Initial and final EQ-5D scores were recorded for MSK participants and WHSS service users. Change in overall EQ-5D score between initial and final assessment was used as the outcome measure and analysed using the change score method as recommended by Lydersen [19,20]. Subgroup analysis was performed stratifying on age, sex, SES, and individual EQ-5D dimension scores for mental health. Trends for age were measured using the Stata® *nptrend* function (StataCorp, College Station, TX). A linear regression analysis was performed to determine the amount of variation in EQ-5D change score which was explained by each predictive variable. The likelihood ratio test was used to examine the statistical significance of an interaction between time to commencement of treatment and mental health status on the EQ-5D change score outcome. Cases with missing EQ-5D data were excluded from the analysis. All analyses were performed using Excel 2007™ (Microsoft Excel, Redmond, WA) or Stata® v.12.1 (StataCorp, College Station, TX).

### Ethics

NHS Lanarkshire deemed ethical approval not to be required, as the study was a secondary data analysis using anonymised routinely collected service data.

## Results

The WHSS database comprised 13 463 unique records, of which 9934 (73.8%) related to musculoskeletal conditions. A total of 3854 (38.8%) of these were for people who were classified as employed and had both initial and final EQ-5D scores, and were therefore eligible for inclusion in the analysis. Overall, the NHS routine MSK dataset comprised records of 23 813 clients, of which 2419 cases had valid paired EQ-5D scores; there were 1054 (43.6%) unique records for employed people which were included in the study. Visual comparison of the demographic characteristics of included and excluded subjects showed little overall difference. Demographic characteristics of the groups included in the study are shown in Table 1.

### EQ-5D change

Overall and in all subgroups, WHSS was associated with a greater degree of change in EQ-5D score than the routine MSK pathway. For WHSS, the greatest benefit was seen in the youngest age-groups, decreasing with age (significant for trend at *p* < 0.001), whilst the trend for age in the MSK participants just failed to achieve statistical significance, *p* = 0.051. WHSS was equally effective for all anatomical sites, whereas the standard MSK pathway showed a greater benefit in back disorders than for lower limb or upper limb and neck problems. For both WHSS and MSK, there was a weak non-significant inverse association with SES, with participants in the lowest socio-economic categories showing the greatest improvement in EQ-5D score (Table 2).

### Enrolment to assessment time

**Figure 1:**
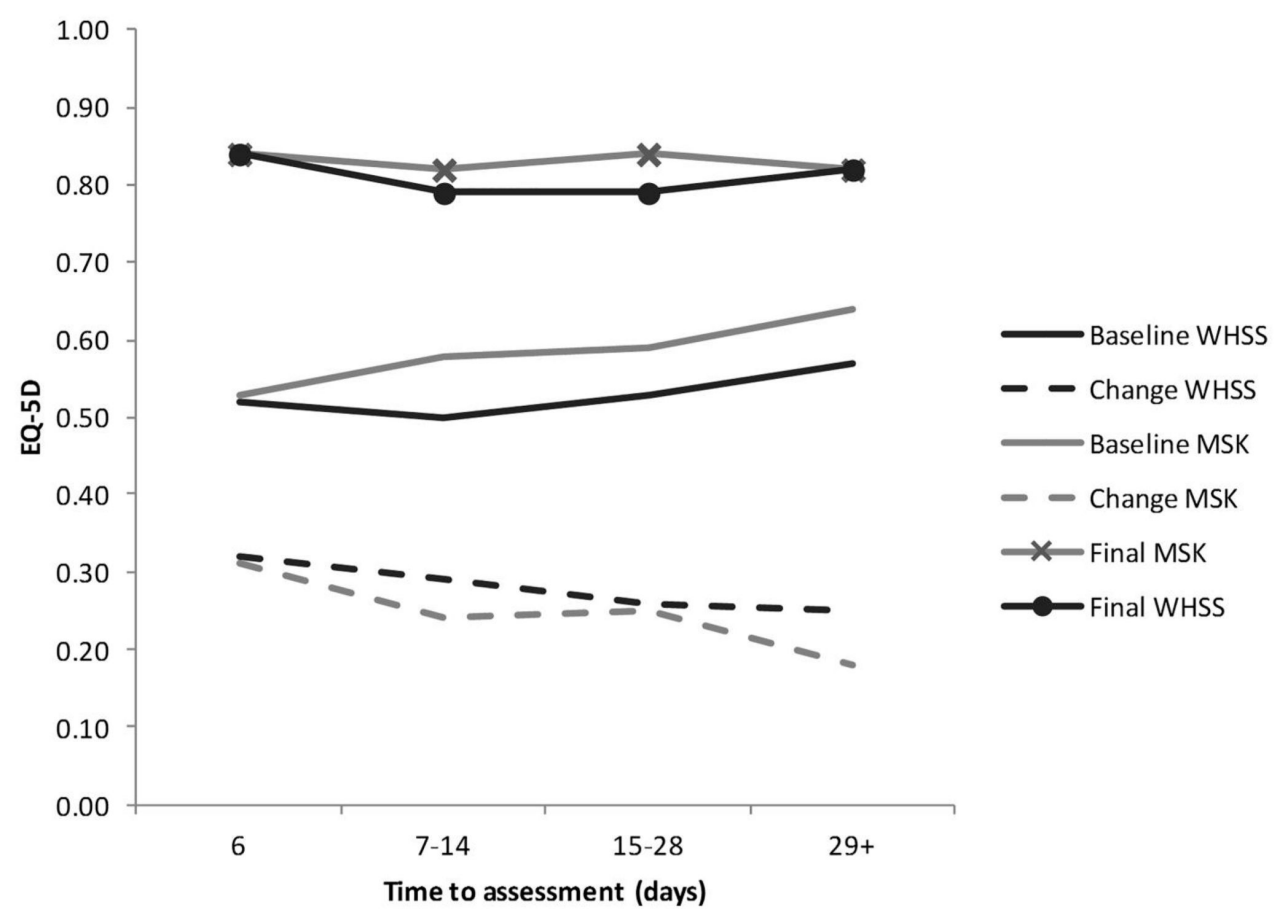
Baseline, change, and final EQ-5D scores for MSK and WHSS by time from enrolment to assessment.

The time interval between enrolment and initial assessment varied between services (MSK/WHSS) and had a major impact on both initial EQ-5D and degree of change achieved. The median interval for WHSS was two days (IQR 0−7 days) whilst for MSK, it was 40 days (IQR 19−63 days). For both MSK and WHSS, longer time from enrolment to assessment was associated with a higher initial EQ-5D and a smaller change (Table 3 and Figure 1).

### Mental health status

**Figure 2:**
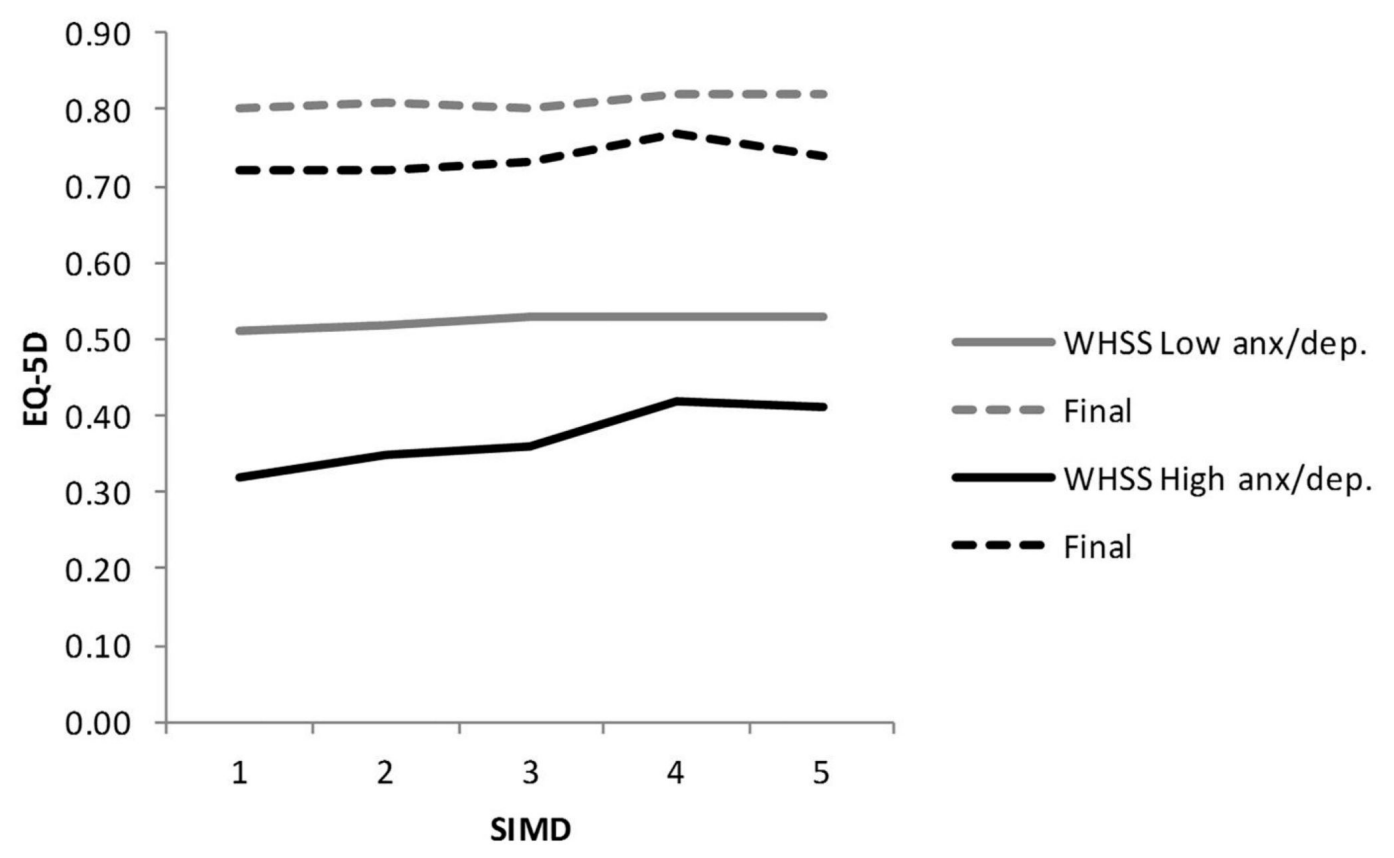
Baseline and final EQ-5D scores for low and medium/high anxiety groups, by socio-economic status (1 = most deprived, 5 = least deprived), WHSS service users (anx/dep: anxiety/depression).

**Figure 3:**
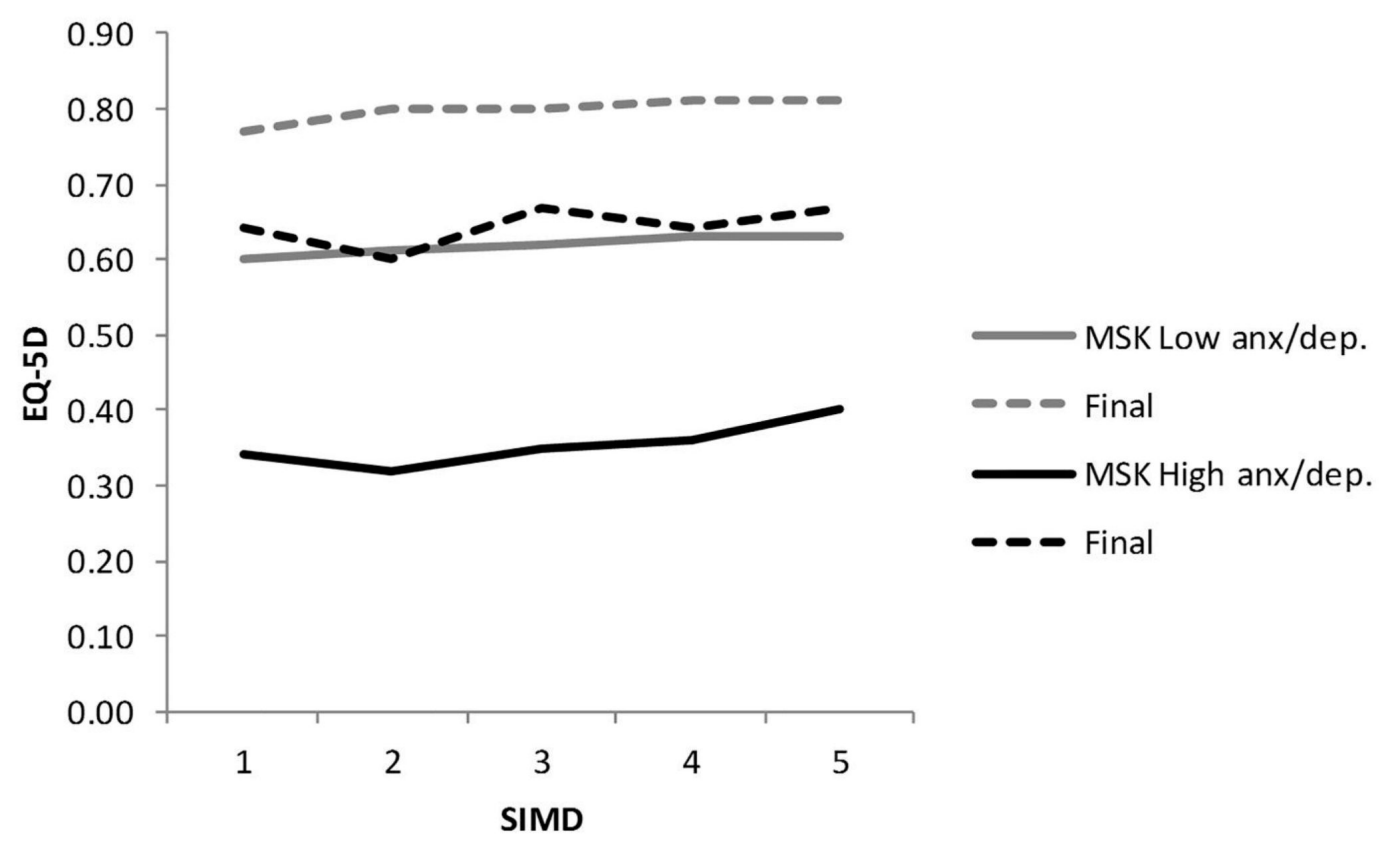
Baseline and final EQ-5D scores for low and medium/high anxiety groups, by socio-economic status (1 = most deprived, 5 = least deprived), MSK service users. anx/dep: anxiety/depression.

In order to explore the impact of mental health on recovery from MSK disorders, the change in overall EQ-5D score was examined stratifying by mental health status (as measured by the anxiety/ depression component of EQ-5D) at baseline, and by SES, and the findings are at Table 4 and Figures 2 and 3. For both WHSS and MSK, a high anxiety/depression score (i.e., poorer mental health) was associated with a lower EQ-5D index at baseline but a greater degree of increase at reassessment, and the effect was greatest, although not statistically significantly so, in the most deprived (SIMD = 1). The case-managed WHSS pathway resulted in a greater increase than the routine MSK pathway, for both medium/ high and low anxiety/depression scores.

Regression analysis confirmed that the increase was highly statistically significant for people with a low anxiety/depression score at baseline, *p* < 0.001, although it was not significant in the higher-scoring group, *p* = 0.087. For service users with low anxiety/depression scores on EQ-5D, the MSK users, whose mean waiting time to start treatment was longer, had higher baseline EQ-5D index at first assessment (mean 0.65) than the WHSS users, who had a shorter wait (mean EQ-5D 0.54). By comparison, there was little difference in baseline EQ-5D index in people with high mental health scores who had experienced the longer MSK waiting period (mean 0.40) compared with 0.39 for WHSS. In people with high levels of anxiety/depression at baseline, WHSS achieved a better final outcome than MSK although high baseline anxiety/ depression scores were consistently associated with lower mean final EQ-5D scores for both the WHSS and MSK pathways (0.76 and 0.73, respectively) than in people with low anxiety/depression baseline scores (0.83 for both).

### Regression analysis

Regression analysis showed that although the overall unadjusted difference in EQ-5D change score, comparing outcomes in WHSS and MSK, was 0.10, 95% CI 0.08−0.12, *p* < 0.001, *R*-squared = 0.02, this was reduced to 0.04, 95% CI 0.02−0.07, *p* = 0.001, *R*-squared = 0.04 after adjusting for age, sex, SES, anxiety/depression level, site of problem, and time to entry to the programme. The only statistically significant contributors to the reduction in effect size were age, time to entry to the programme, and mental health status at baseline. There was a highly significant interaction (*p* = 0.001) between EQ-5D change score and mental health status at baseline, and also between EQ-5D change score, time to commencement of treatment, and mental health status.

### User satisfaction - WHSS

The 20 WHSS service users (0.5% of those included in the study) who demonstrated the greatest gain in EQ-5D score all had EQ-5D scores <0 at baseline. Fifteen (75%) considered their condition fully resolved and five (25%) partially resolved. Seventeen (85%) recorded their satisfaction as “excellent”, one (5%) rated it as ”good”, and two (10%) did not have this measure recorded. Free text user comments noted, for example, that:

- Physiotherapy was effective and the care manager provided helpful advice.
- Self-employed people valued prompt access to treatment.

In comparison, 20 (0.5%) WHSS service users who demonstrated a reduction in EQ-5D (indicating a worsening of their condition) nonetheless also showed a high level of satisfaction with the service, with almost all for whom this was recorded rating it “excellent” (10 (50%)) or “good” (9 (45%)), despite 90% reporting that their health condition was unresolved. Only one (5%) of this group rated the service as “poor”. The narrative comments from users experiencing a poor outcome reflected the complexity of issues encountered, and few were entirely negative, for example:

- Poor workplace management attitudes to support services such as occupational therapy could hinder recovery.
- Even if the service did not achieve its primary aim of a return to work, the prompt access to physiotherapy and support of the case manager were appreciated.

Another user whose score did not improve nonetheless gave a positive assessment, reporting that the service had helped in coming to terms with their unsuitability for their present employment and encouraging them to seek alternative work which was more appropriate to their condition.

Detailed satisfaction data and narrative comments were not available for MSK service users.

## Discussion

Our results demonstrate that whilst both the NHS MSK service and referral to WHSS were associated with improved overall outcomes (as measured by change in EQ-5D) in people with musculoskeletal disorders who are in current employment, the case-managed approach of WHSS achieved significantly greater change. The benefit was greatest in those with the poorest mental health status at baseline. Those who were in the most deprived socio-economic categories also showed evidence of greater benefit although this did not achieve statistical significance. The shorter pathway from enrolment to assessment afforded by WHSS, the different age profiles of the groups, and differences in mental health status at enrolment all contributed to the findings. Earlier entry to the programme was associated with a lower baseline score than those who started treatment at a later point in the recovery pathway, but overall, the final EQ-5D scores were similar for both services.

Among people with low anxiety/depression scores at baseline (i.e., better mental health), the MSK users had a higher EQ-5D index at first assessment than the WHSS users. By contrast, for those who had high baseline anxiety/depression scores (i.e., poorer mental health), there was no difference in EQ-5D index between MSK and WHSS users at first assessment. As there is no plausible reason for a systematic difference between WHSS and MSK users at the time of enrolment, and the MSK users had a longer wait to treatment than the WHSS users, this suggests spontaneous improvement during the longer waiting period in the absence of mental health issues. For those with poor mental health at enrolment, however, no improvement during the waiting time between enrolment and the start of treatment could be inferred.

For the MSK pilot, there was little difference in level of improvement for any category of age or gender. However for WHSS, those who achieved the greatest benefit were young (<40 years of age) people in the lowest socio-economic categories, and those with higher anxiety/depression scores. Although we had no data on cause of problem, it is possible that work-related injuries predominated in young people in the lower socio-economic groups; especially in manual workers, musculoskeletal disorder would have had a major impact on ability to remain at work [21].

Our findings therefore show not only that the case-managed biopsychosocial approach is associated with significantly better results, in people who are currently in employment, but also that those who are most disadvantaged through concurrent mental health problems gain the greatest benefit from case-management. We have therefore confirmed the findings of a number of researchers [11,22] in supporting the role of case-management, and we have added a further dimension by demonstrating that participants with musculoskeletal problems who had the poorest mental health at baseline showed the greatest gains in overall health with the biopsychosocial case-management approach. This finding is consistent with the identification, in a comprehensive systematic review, that not only are biopsychosocial factors an important prognostic indicator in recovery from MSK disorder, but also that it is the psychosocial component which predominates [8]. It is also consistent with the findings of Carnide et al., who showed that persistent depressive symptoms are associated with poor return to work outcomes [9], and thereby contributes to the evidence base showing that mental health status at baseline is an important factor in recovery from MSK disorder. This is an important finding as it has been shown that around 20% of patients with chronic back pain have co-morbid major depression [5]. Huxley et al. showed an improvement in quality of life with case-management in depressive illness which was most marked in those with more severe disease at baseline [23], whilst Boot et al. found that mental health was a predictor for return to work in people with physical co-morbidity [24].

We have also shown that earlier entry to the programme contributes to the effectiveness of case-management, although some of this finding may be explained by natural recovery, as demonstrated by Sinclair et al. [25]. Few longitudinal studies have examined natural recovery pathways in musculoskeletal disorder, but there is some evidence that better recovery is associated with younger age [26], which is consistent with our findings.

Perhaps counter-intuitively, we did not find SES alone to be a statistically significant predictive variable. Notwithstanding, Figures 2 and 3 demonstrate a gradient in baseline EQ-5D from low to high SIMD in service users with high/medium anxiety depression scores, with the greatest improvement in the most deprived category, despite lack of statistical significance. Further, SES is highly correlated with mental health status and we did observe a highly significant interaction between mental health status and SES. Previous studies which have reported SES to be predictive have generally been in the context of a more complex regression analysis including for example racial factors and legal representation [27]. The authors noted the complexity of the association between SES, health, and healthcare outcomes, and this is further emphasised by the findings of Boot et al. in relation to comorbidities, mental health, and household income [24]. There is a well-recognised inverse relationship between SES and health [28], and there is evidence that poorer healthcare outcomes are influenced by selection bias as those from more deprived back-grounds are more likely to present with advanced disease [29]. Individuals with lower SES are more likely to have concomitant social problems because of life circumstances [30] and for these, the biopsychosocial approach of case-management is likely to be more effective than the more traditional medical model of care utilised by the MSK pathway [31].

By facilitating timely access to appropriate healthcare, case-management addresses the important biopsychosocial barrier to recovery, and thereby provides the greatest benefit to the most disadvantaged, contributing to reducing health inequalities. Since manual workers are both predominantly drawn from the lower end of the socio-economic spectrum, and are less likely than sedentary workers to be able to continue working in the presence of a musculoskeletal disorder, the economic benefits of improving recovery and hence return-to-work time through case-management are clear.

### Strengths and limitations

The principal strength of this study was the existence of large sets of routinely collected data which facilitated the comparison of the two groups, one of which was based on an existing active case-management service. A weakness of the study was the heterogeneity of the datasets, whereby the EQ-5D score was the only variable common to both which had utility as a valid outcome measure. This inevitably limited the range of analyses which could be performed. There were demographic differences between the WHSS and MSK service users; WHSS service users were more likely to be younger and employed, whereas a large number of MSK users were older and retired. We mitigated the effects of the difference in population demographics by restricting eligibility for inclusion to those who were in current employment, by stratifying on age in the analyses, and by using regression analysis to examine the impact of confounders. Nonetheless, the model was limited in its ability to explain the differences between the groups and there may be other explanatory factors which were not recorded. As the demographic characteristics of those with and without EQ-5D scores were similar, the assumption has been made that the treatment outcomes of those with EQ-5D scores were representative of the entire group. Only 44% of MSK service users with valid EQ-5D data were eligible for inclusion, owing to their employment status. This reduced the numbers eligible for inclusion and therefore reduced the statistical power of the analyses. The use of change scores has been disputed as being less reliable than the component variables [32]; however, Overall and Woodward have demonstrated a “reliability paradox” whereby the method remains valid in assessing the significance of treatment-induced change, even in the presence of low reliability of scores [19].

In conclusion, whilst timely case-management of MSK disorder can deliver benefits to all participants, the overall health gain is greatest in people who have poor baseline mental health status. Case-management therefore makes an important contribution to reducing health inequalities in this vulnerable group, who may otherwise be at highest risk of long-term sickness absence and worklessness. Case-management should form an important component of strategies to minimise long-term sickness absence due to MSK disorders, with particular emphasis on services for people with poor mental health.

## Figures and Tables

**Table 1:** Demographic characteristics of the groups, employed people only.

		WHSS *n* (%)	MSK *n* (%)	*p* Value
Total		3854 (100.0)	1054 (100.0)	
Sex	Men	1994 (51.7)	441 (41.8)	
	Women	1842 (47.8)	596 (56.6)	<0.001
	Missing data	18 (0.5)	17 (1.6)	
Age group	<30 years	399 (10.4)	128 (12.1)	
	30−39 years	731 (19.0)	131 (12.4)	
	40−49 years	1196 (31.0)	308 (29.2)	
	50−59 years	1142 (29.6)	356 (33.8)	
	≥60 years	386 (10.0)	131 (12.4)	<0.001
Presenting complaint	Back	1306 (33.9)	331 (31.4)	
	Lower limb	1109 (28.8)	328 (31.1)	
	Upper limb/neck	1439 (37.3)	395 (37.5)	0.214
SIMD quintile	1	547 (14.2)	201 (19.9)	
	2	728 (18.9)	287 (27.2)	
	3	809 (20.1)	230 (21.8)	
	4	961 (22.3)	198 (18.8)	
	5	909 (23.6)	108 (10.3)	<0.001
	Missing data	−	21 (2.0)	

(−) No cases. *p* Value for chi-squared.

**Table 2:** Change in EQ-5D score.

		WHSS *n*= 3854	95% CI	MSK *n*= 1054	95% CI	*p* Value
Baseline EQ-5D score		0.52	0.51−0.53	0.62	0.61−0.63	<0.001
*Change in score* Overall		0.30	0.30−0.31	0.20	0.19−0.22	<0.001
Sex	Men	0.30	0.29−0.31	0.21	0.19−0.23	<0.001
	Women	0.31	0.30−0.32	0.20	0.19−0.22	<0.001
Age group	<30 years	0.32	0.30−0.35	0.22	0.17−0.26	<0.001
	30−39 years	0.32	0.30−0.34	0.22	0.18−0.25	<0.001
	40−49 years	0.31	0.29−0.32	0.19	0.17−0.22	<0.001
	50−59 years	0.30	0.28−0.31	0.21	0.19−0.23	<0.001
	≥60 years	0.28	0.26−0.31	0.19	0.15−0.22	<0.001
Presenting complaint	Back	0.31	0.29−0.32	0.23	0.20−0.25	<0.001
	Lower limb	0.30	0.28−0.31	0.22	0.20−0.24	<0.001
	Upper limb/neck	0.31	0.29−0.32	0.17	0.15−0.19	<0.001
SIMD quintile	1	0.31	0.29−0.33	0.22	0.19−0.24	<0.001
	2	0.32	0.30−0.34	0.20	0.18−0.22	<0.001
	3	0.29	0.27−0.31	0.21	0.18−0.24	<0.001
	4	0.30	0.29−0.32	0.20	0.17−0.23	<0.001
	5	0.30	0.28−0.32	0.19	0.15−0.23	<0.001

CI: confidence interval; SIMD: Scottish Index of Multiple Deprivation (1 = most deprived, 5 = least deprived). *p* Value for two-sample Student’s *t* test comparing WHSS and MSK.

**Table 3:** Change in EQ-5D by time from enrolment to assessment, by service.

Service	Enrolment to assessment	*n*	Mean EQ-5D baseline	Mean EQ-5D change	95% CI for change	Mean EQ-5D final	*p* Value for difference between WHSS and MSK
WHSS	≤6 days	2802	0.52	0.31	0.30−0.32	0.83	−
	7−14 days	729	0.50	0.29	0.27−0.31	0.79	−
	15−28 days	246	0.53	0.26	0.23−0.29	0.79	−
	≥29 days	72	0.57	0.24	0.17−0.30	0.81	−
MSK	≤6 days	50	0.53	0.31	0.25−0.37	0.84	0.830
	7−14 days	150	0.58	0.23	0.20−0.26	0.81	0.025
	15−28 days	177	0.59	0.24	0.22−0.28	0.83	0.726
	≥29 days	599	0.64	0.19	0.17−0.20	0.83	0.031

CI: confidence interval. 95% CI for change in EQ-5D index: *p* value for two-sample Student’s *t* test.

**Table 4:** Change in EQ-5D score by mental health status.

EQ-5D score for anxiety/depression	WHSS				MSK			95% CI	*p* Value
	*n*	Baseline EQ-5D index	Change in EQ-5D index	95% CI	*n*	Baseline EQ-5D index	Change in EQ-5D index		
1 or 2 (low)	3270	0.54	0.29	0.28−0.30	916	0.65	0.18	0.17−0.20	<0.001
3, 4, or 5 (med-high)	563	0.39	0.37	0.35−0.39	138	0.40	0.33	0.29−0.38	0.087

CI: confidence interval. 95% CI for change in EQ-5D index: *p* value for two-sample Student’s *t* test comparing WHSS and MSK.

## Data Availability

The data used in this study are not currently available for sharing.

## Implications for Rehabilitation

1. Musculoskeletal disorders are a major cause of inability to work.
2. Case-management is effective in helping people with musculoskeletal disorders to return to work.
3. People who have the poorest mental health are likely to gain the greatest benefit from case-management of their musculoskeletal disorders.
